# Longitudinal analysis of mRNA transcripts and plasma proteins to define a biomarker associated with lupus disease activity

**DOI:** 10.1186/ar3937

**Published:** 2012-09-27

**Authors:** M Olferiev, W-T Huang, KA Kirou, E Gkrouzman, D Lundsgaard, KS Frederiksen, J Fleckner, MK Crow

**Affiliations:** 1 Mary Kirkland Center for Lupus Research, Hospital for Special Surgery, New York, NY, USA; 2 Novo Nordisk, Copenhagen, Denmark

## Objective

Lupus, a chronic autoimmune disease, is characterized by a variable clinical course, with periods of active disease. Identification of a biomarker or biomarker panel associated with clinical disease activity would be useful for disease management, assessment of response to therapeutic intervention in practice or clinical trials, and might suggest cellular or molecular targets for future therapies. To identify biomarkers that reflect lupus disease activity, we assessed longitudinal clinical, gene expression and proteomic data from SLE patients.

## Methods

One hundred and sixty-nine RNA extracts from PBMC and plasma samples were collected longitudinally (up to 3 years) from 23 SLE patients and five healthy donors (HD), and SLEDAI and BILAG scores were recorded. All SLE patients fulfilled ACR criteria for the disease. PBMC mRNA profiles for each visit were established using Affymetrix GeneChips. A panel of proinflammatory cytokines was evaluated using Multi-Analyte Profiling technology (Rules-Based Medicine, Austin, TX, USA). Longitudinal data analysis was performed using R (R Development Core Team) and the R packages lme4 and languageR. Data were analyzed using linear mixed-effects (LME) models.

## Results

K-mean cluster analysis was first used to identify groups of gene transcripts that fluctuate in relation to disease activity, and representative transcripts were selected from each cluster. Thirteen plasma factors were identified as significantly increased in SLE patients compared with HD, and 14 plasma factors were significantly associated with disease activity. LME analysis was applied to the dataset to identify those transcripts and plasma factors that best define clinical disease activity. Statistical correlation with disease activity for this biomarker panel was compared with traditional measures of disease activity.

## Conclusion

A combination of mRNA transcripts and plasma factors, when assessed as a panel, shows a high correlation with clinical disease activity in patients with SLE. Validation of this biomarker panel in an extended patient group may provide support for measurement of these transcripts and proteins as an informative correlate of disease activity and a tool for patient management.

